# SARS-CoV-2 Associated With Pneumothorax: A Case Report and Literature Review

**DOI:** 10.7759/cureus.12191

**Published:** 2020-12-20

**Authors:** Islam Younes, Mahsa Mohammadian, Sherif Elkattawy, Zamir Singh, Michael L Brescia

**Affiliations:** 1 Internal Medicine, Rutgers New Jersey Medical School/Trinitas Regional Medical Center, Elizabeth, USA; 2 Internal Medicine, Rutgers-New Jersey Medical School/Trinitas Regional Medical Center, Elizabeth, USA; 3 Internal Medicine, St. George's University School of Medicine, Elizabeth, USA; 4 Critical Care, Rutgers-New Jersey Medical School/Trinitas Regional Medical Center, Elizabeth, USA

**Keywords:** covid 19, pneumothorax

## Abstract

SARS-CoV-2 has created universal disarray since its outbreak in 2019. Emergent measures were taken worldwide to mitigate the morbid outcomes of the pandemic. Multiple organ systems have been shown to be negatively impacted secondary to the heightened inflammatory response to the novel virus. In this report, we focus on the respiratory system. The novel virus impact on the respiratory system has been well documented, leading to acute respiratory distress syndrome. Here, we present a case of a patient with no risk factors for pneumothorax (smoking, underlying lung disease, prior history of pneumothorax, age, family history) who was found to be SARS-CoV-2 positive and developed a significant pneumothorax requiring transfer to the intensive care unit.

## Introduction

Since December 2019, the world has experienced one of the most morbid pandemic crises known as COVID-19 caused by SARS-CoV-2. This novel virus has led to severe social, medical, and economic implications worldwide. Clinical manifestations are diverse in severity with bilateral pneumonia as the main finding in hospitalized patients. We report a case of polymerase chain reaction (PCR)-confirmed COVID-19 in a 67-year-old Hispanic male with a past medical history of hypertension who developed bilateral spontaneous pneumothorax with no known risk factors and without any positive pressure ventilation.

## Case presentation

**Figure 1:**
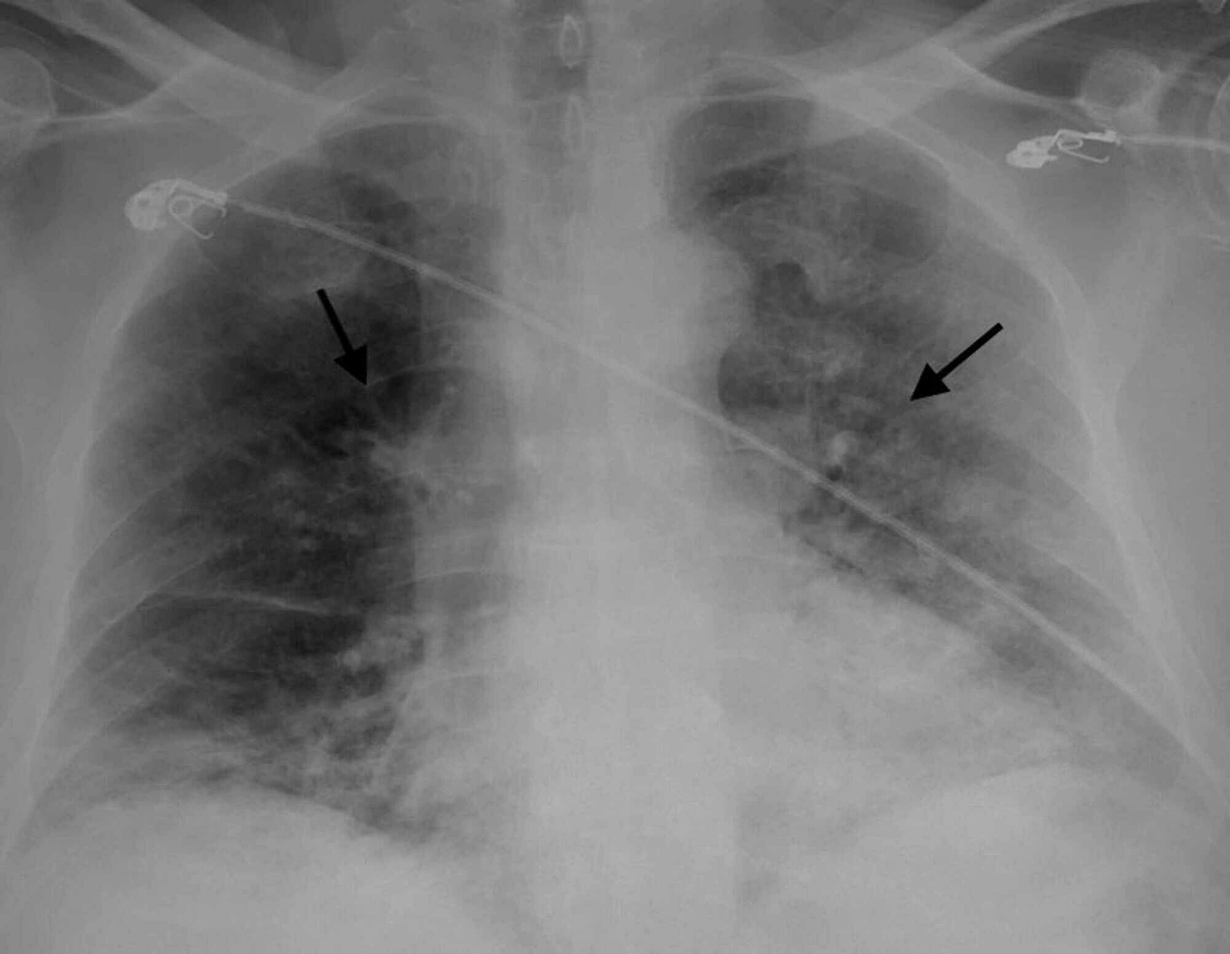
Chest X-ray shows bilateral infiltrates as shown by arrows

A 67-year-old Hispanic male with a past medical history of hypertension presented to the emergency department with a one-week history of fever, cough, generalized malaise and shortness of breath. The patient denied any tobacco smoking history. The patient was afebrile, with SpO2 85% on room air, tachypneic with RR 24, BP 137/80, and HR 75. Lung examination was significant for bilateral scattered crackles. Initial chest X-ray showed bilateral infiltrates as seen in Figure 1.

**Figure 2:**
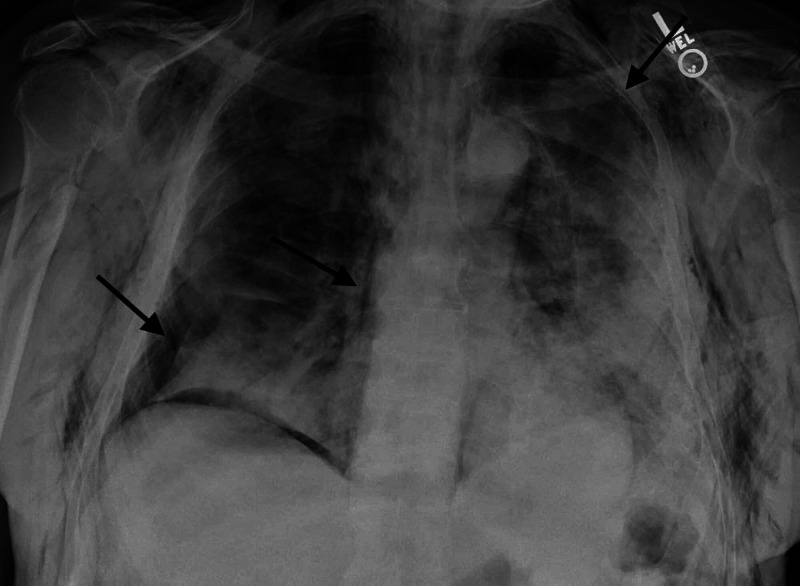
Chest X-ray shows bilateral pneumothoraces as well as pneumomediastinum as shown by arrows

Labs were unremarkable except for WBC 7.8 k/ul (4.8-10.8 K/UL) with mild lymphopenia. The patient tested positive for COVID 19 and was admitted to the medical floors for further management. The patient was started on dexamethasone 6 mg daily, remdesivir 200 mg once and then 100 mg daily for nine days as well as prophylactic enoxaparin. He was also started on nasal cannula 4LPM for oxygen support. He initially showed improvement regarding his oxygen saturation and tachypnea on the nasal cannula. However, during the hospital stay, the patient acutely deteriorated with subcutaneous crepitations noted on his neck and anterior bilateral chest. Repeat chest X-ray showed bilateral pneumothorax, more so on the right, and subcutaneous emphysema in the upper chest and neck as seen in Figure 2.

**Figure 3:**
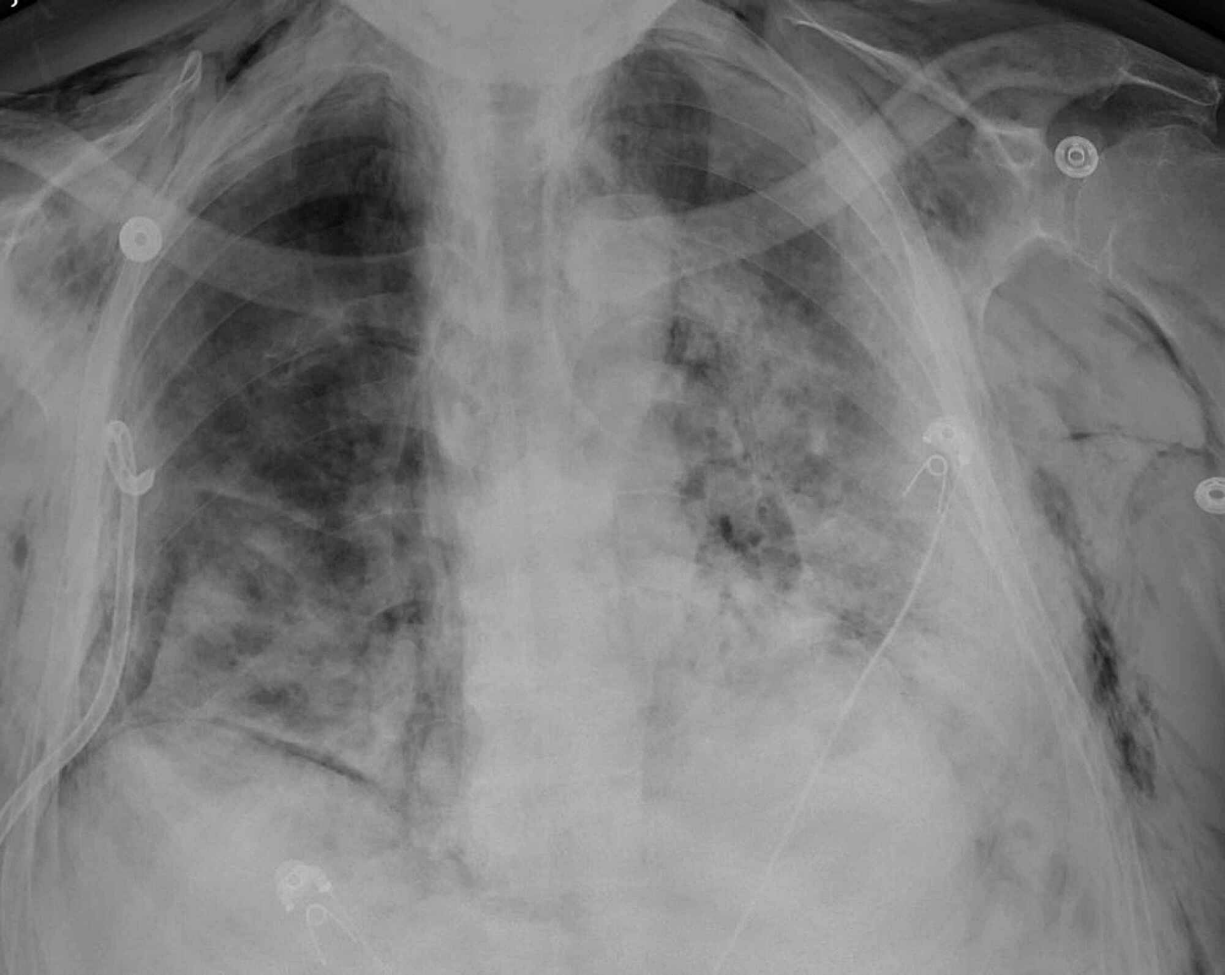
Chest X-ray shows significant improvement of right-sided pneumothorax s/p right tube thoracostomy

Pigtail was placed on the right side as seen in Figure 3.

The patient was transitioned to a high-flow nasal cannula and transferred to the ICU for closer monitoring. Respiratory status continued to deteriorate and the patient eventually required invasive mechanical ventilation. Prone positioning was attempted to no avail. During the intensive care unit stay, the patient had a cardiac arrest with failure of resuscitation in the setting of acute respiratory distress syndrome from COVID 19 pneumonia after 18 days of hospitalization.

## Discussion

Pneumothorax and pneumomediastinum have been reported in patients with COVID-19. Although this is seen in a small number of patients, the relationship between COVID-19 and pneumothorax is unclear. Spontaneous pneumothorax is a known complication of the severe acute respiratory syndrome (SARS), which was caused by SARS-CoV-1, with an incidence of 1.7% in hospitalized patients [1]. Given the potential consequences of pneumothorax in hospitalized patients, it is important to further assess the frequency of occurrence in patients with COVID-19 in order to establish preventative measures and optimize treatment regimens.

Pneumothorax is classified as either primary or secondary. A primary pneumothorax occurs in the absence of notable lung pathology and does not have an obvious cause. A secondary pneumothorax is seen in the setting of existing lung pathology. The diagnosis of pneumothorax involves imaging in the form of a chest X-ray or a computed tomography (CT) scan, with the latter being the gold standard. The most common findings on a CT scan in patients with COVID-19 include bilateral and peripheral ground-glass opacities, as well as consolidative pulmonary opacities. There is a temporal link between CT findings and the stage of the disease, with 56% of patients with the early disease having normal findings [2]. The further on from disease-onset, the more substantial and frequent the findings on a CT-scan. These findings include greater lung involvement, the reverse-halo sign, linear opacities and a crazy-paving pattern [3]. 

Current theories regarding the pathogenesis of pneumothorax in non-ventilated patients with COVID-19 state that diffuse alveolar injury due to a COVID-19 infection increases the risk of alveolar rupture, which can result in pneumothorax [4]. This is supported by the fact that the most common CT finding in patients with COVID-19 pneumonia is ground-glass opacities, which can be attributed to alveolar swelling and alveolar septal inflammation secondary to infection [4]. The common risk factors for pneumothorax include smoking, genetic disorders, family history and tall stature. Our patient denied any smoking history, had no known significant family history and was of average height. Moreover, the use of ventilators in the management of these patients is an independent risk factor for the development of pneumothorax. The treatment of this pathology is dependent on several factors, from the size of the pneumothorax, its mechanism, the treating physician and even patient preference [3,5].

In patients with COVID-19, mechanical ventilation seems to be a major risk factor for the development of pneumothorax [5]. It can result in the overdistension of alveoli, which in the case of COVID-19 can exacerbate the existing damage resulting in a pneumothorax. There is a high risk of progression to a tension pneumothorax in these patients, with immediate tube thoracostomy required to prevent this. Given that up to two-thirds of patients with COVID-19 who need admission to the critical care unit require mechanical ventilation [6], treating physicians should be aware of this complication, as well as the recommended management. With only two-thirds of patients with COVID-induced pneumothorax surviving, it is important to note that certain factors are associated with a poor prognosis, such as age greater than 70 and associated acidosis [1]. With the limited information available on these patients and the novelty of this virus, it is essential to further educate clinicians so that they may identify the higher-risk populations, such as those at risk for pneumothorax. This will enable them to provide more immediate and efficient treatment in accordance with established practice guidelines, aiding in the efforts to minimize the morbidity and mortality of this disease.

## Conclusions

SARS-CoV-2 has led to worldwide precautionary measures when out in public given morbid outcomes of acquiring the novel virus. Multiple complications to those inflicted with the virus have been well documented in the literature. Our report here brings attention to pneumothorax as a deadly complication and the importance of recognizing patients at high risk and managing accordingly.
